# XLH Matters 2024: expert insights and practical tools for enhancing care of people living with X-linked hypophosphataemia

**DOI:** 10.1186/s13023-025-03930-x

**Published:** 2025-09-18

**Authors:** Lothar Seefried, Ali S. Alzahrani, Carsten A. Wagner, Damian Eade, Danilo Fintini, Dieter Haffner, Hasan Frookh Jamal, Judith S. Bubbear, Laura Guazzarotti, Moira S. Cheung, Noina Abid, Patrícia Costa-Reis, Rui Ferreira Santos, Signe Sparre Beck-Nielsen, Agnès Linglart

**Affiliations:** 1 Orthopedic Institute, König-Ludwig Haus, University of Würzburg, Würzburg, Germany; 2 Department of Medicine, King Faisal Specialist Hospital and Research Centre, Riyadh, Saudi Arabia; 3 Institute of Physiology, University of Zurich, Zurich, Switzerland; 4 Lumanity, Insight – Social Media Insight and Analytics, London, UK; 5 Endocrinology and Diabetology Unit, Bambino Gesù Children Hospital, IRCCS, Rome, Italy; 6 Department of Pediatric Kidney, Liver, Metabolic and Neurological Diseases, Hanover Medical School, Hanover, Germany; 7 Department of Medicine, Salmaniya Medical Complex, Government Hospital, Ministry of Health, Salmaniya, Kingdom of Bahrain; 8 Centre for Metabolic Bone Disease, Royal National Orthopaedic Hospital, Stanmore, UK; 9 Pediatric Endocrinology Unit Department of Woman and Child Health, University of Padua, Padua, Italy; 10 Great Ormond Street Hospital, NHS Foundation Trust, London, UK; 11 Royal Belfast Hospital for Sick Children, Belfast, Northern Ireland; 12 Pediatric Nephrology and Kidney Transplant Unit, Hospital de Santa Maria, Faculdade de Medicina, Universidade de Lisboa, Lisbon, Portugal; 13 Evelina London Children’s Hospital/Guy’s and St Thomas’ NHS Foundation Trust, London, UK; 14 Centre for Rare Diseases, Aarhus University Hospital and Department of Clinical Research, Aarhus University, Aarhus, Denmark; 15 AP-HP, Endocrinology and Diabetology for Children, Bicêtre Paris Saclay Hospital, Paris Saclay University, Le Kremlin-Bicêtre, France

## Introduction to XLH Matters 2024

### Background

X-linked hypophosphataemia (XLH) is a rare, genetic, lifelong disorder caused by pathogenic variants in the phosphate-regulating endopeptidase homologue X-linked *(PHEX)* gene [1, 2]. Loss-of-function mutations in the *PHEX* gene lead to increased levels of fibroblast growth factor 23 (FGF23) causing renal phosphate wasting and low calcitriol levels due to impaired production and accelerated degradation of calcitriol [3]. In most cases, XLH is inherited from an affected parent; however, in 20–30% of cases, XLH is assumed to arise from de novo mutations [4–6].

Clinical manifestations of XLH typically emerge in early childhood, often becoming apparent when infants begin to bear weight [7]. The chronic hypophosphataemia and calcitriol deficiency lead to rickets in children, marked by impaired bone mineralisation, defective calcification of growth plate cartilage, delayed endochondral ossification and slowed growth velocity [7, 8]. Beyond rickets, these children commonly experience lower extremity deformities, reduced growth, impaired muscle function and pain. Dental abscesses are also prevalent due to impaired mineralisation of both dentin and enamel [8]. Craniosynostosis, skull suture ossification before 5 years of age, can result in altered head size/circumference and elevated intracranial pressure [9]. These challenges have a negative impact on emotional wellbeing, with affected children often displaying lower school attendance and participation [7, 8].

Typical characteristics of XLH in adults include osteomalacia, pseudofractures, musculoskeletal pain, stiffness, osteoarthritis and enthesopathies, all resulting in impaired physical functioning and mobility [10]. Unresolved childhood disease can add to the adult burden of comorbidities with lower limb deformities and impaired mobility [8]. In addition, musculoskeletal complications are common in adults living with XLH, such as osteoarthritis, enthesopathy and spinal stenosis, beginning as early as 20 years old and accumulating with age [11]. Other complications of XLH in children and adults include hyperparathyroidism, obesity and impaired hearing as early as age 11 years [5, 8, 9].

### XLH Matters 2024

XLH Matters is a two-day annual networking event for healthcare professionals (HCPs) who manage children and adults living with XLH across a range of specialties and geographical regions. The event began as a virtual meeting in 2021, followed by in-person events in 2022, 2023 and 2024. Insights from the XLH Matters 2022 and 2023 meetings have been published [12, 13]. The 2024 meeting welcomed 122 HCPs from 27 countries, including paediatric and adult endocrinologists, nephrologists, rheumatologists, general paediatricians, orthopaedic surgeons and nurse specialists. Key objectives of XLH Matters 2024 are listed in Box 1.

**Box 1:** Key objectives of the XLH Matters 2024 meeting

1) Provide an update on XLH developments and their impact on clinical practice 2) Identify optimal approaches to collaborate within an XLH multidisciplinary team (MDT) 3) Evaluate the impact of previous XLH Matters meetings on clinical practice 4) Discuss effective monitoring strategies across the lifespan of people living with XLH 5) Examine strategies to treat and manage complex cases of children and adults living with XLH	

This supplement aims to provide a comprehensive summary of the key insights from plenary presentations, workshop sessions and faculty panel discussions at XLH Matters 2024. It highlights the latest advancements in XLH care, emphasising the importance of multidisciplinary collaboration, effective monitoring strategies and prioritising the patient experience. The meeting addressed challenges and knowledge gaps in XLH management, offering practical solutions to enhance monitoring practices and foster collaboration with multidisciplinary experts. The intention is to advance clinical practice, improve patient outcomes and enhance the quality of life for people living with XLH.

## Latest news from the XLH field

As research into XLH continues to expand, new insights are emerging that enhance our understanding of its pathophysiology diagnosis and treatment. Recent studies have focused on the role of FGF23 as a diagnostic marker and the efficacy of burosumab treatment in children with XLH [14–16].

FGF23 measurement may serve as a valuable diagnostic tool for investigating phosphate disorders. However, the normal reference values for intact or C-terminal FGF23 can vary significantly in children depending on the methodology used and the child’s age range. To address this variability, a cross-sectional study evaluated the normal reference values according to chronological age and pubertal development in healthy infants, children and adolescents [14]. FGF23 levels in 282 children and adolescents, including 20 individuals with XLH aged 10.2 ± 5.6 years, were compared with healthy controls. The intact FGF23 levels were significantly elevated in individuals with XLH compared with age-adjusted healthy controls (p < 0.0001) and showed an inverse correlation with serum phosphate concentration (p < 0.01). Notably, infants with XLH had the highest plasma concentrations of intact FGF23 among the patient groups. These findings underscore the importance of FGF23 as a critical diagnostic marker in phosphate-wasting disorders [14].

Burosumab was first shown to be effective in improving phosphate metabolism and promoting rickets healing in children living with XLH in early clinical trials [15]. Building on this, additional and confirmatory evidence supporting the use of burosumab in children with XLH was demonstrated in the study by Ward LM, et al., which reported improvements in rickets healing, phosphate metabolism and growth over 88 weeks of treatment [16, 17]. In the extension period (Weeks 64–88), 21 children continued burosumab treatment Q2W (n = 6) or crossed over from oral phosphate and active vitamin D to burosumab, starting at 0.8 mg/kg Q2W (n = 15). Improvement in rickets healing, parameters of phosphate metabolism and growth were observed from baseline to Week 88 among those who continued or switched to burosumab. No new safety signals were identified [16].

Other recent advances in the understanding of XLH diagnosis and pathophysiology are outlined in Box 2.

**Box 2:** Selected notable advances in the diagnosis and pathophysiology of XLH

• *Patients living with XLH have altered muscle geometry* [18]: The lower limb muscles of 11 patients with XLH were analysed using 3D reconstruction with magnetic resonance imaging (MRI) and compared with those from 15 healthy controls (mean age: 10 years) in a prospective study. The analysis revealed significantly reduced mean normalised muscle lengths in the femoral biceps, gluteus maximus, rectus femoris and tensor fasciae latae among people living with XLH compared with healthy controls, whereas the medius/minimus gluteus muscles did not show significant differences • *Adults living with XLH have reduced muscle size and strength* [19]: A case–control study characterised muscle deficits and assessed intramuscular phosphate metabolites in adults living with XLH at resting state (aged 18–65 years; n = 13) compared with age/sex/body weight-matched controls (n = 13). There was a higher phosphocreatine to inorganic phosphate (PCr/Pi) ratio in adults with XLH compared with those in the controls (p = 0.023). Other metabolites were comparable between the two groups • *Improved height in French people living with XLH may reflect earlier diagnosis and improved therapies* [20]: People living with XLH typically present with short stature and final heights below the average of reference populations. A retrospective analysis of the natural evolution of height in 398 French patients with XLH across three birth cohorts (1950–1975, 1976–2000 and 2001–2006) indicated significant improvement in final height in French patients with XLH born after 2001, with a gain of almost 1 standard deviation (SD). This may be attributed to earlier diagnosis and improved therapies • *Direct neutralisation of FGF23 improves dental manifestations in a mouse model of XLH *[21]: Burosumab treatment (4 or 16 mg/kg) restored dentin/cementum volume and corrected the enlarged pulp volume in *Hyp* mice, with the higher concentration resulting in a rescue similar to wildtype (WT) levels • Testing the role of XLH, FGF23 and calcitriol on calcification of the Achilles tendon in mouse models of XLH [22]: In the absence of calcitriol, WT mice exhibited enthesopathy similar to that seen in *Hyp* mice. However, the deletion of FGF23 in *Hyp* mice prevented the development of enthesopathy. Early restoration of 1,25-dihydroxyvitamin D (calcitriol) (1,25[OH]_2_D) in mice models prevented development of enthesopathy but did not reverse it. These results show that 1,25(OH)_2_D signalling may play a role in the development of enthesopathy in XLH	

## Advancing healthcare through XLH Matters

### The impact of XLH Matters on clinical practice

At XLH Matters 2024, three clinicians shared their experiences of optimising clinical practice after attending XLH Matters 2023 (Table 1).

**Table 1:** Changes made to clinical practice at three centres following attendance at XLH Matters 2023

Centre of practice	Changes implemented after XLH Matters 2023
Centre 1: Italy	• Developed a **healthcare transition programme** with multidisciplinary and psychological support for adolescents transitioning to adult care • Explored **optimal methods for evaluating growth outcomes** in children living with XLH o Identified the **sitting height/standing height (SitHt/Ht) ratio** as the best method to track segmental growth differences in children and adolescents living with XLH; patients experience greater trunk growth compared with leg growth, particularly during puberty, leading to an increased SitHt/Ht ratio [23] o Trunk lengthening was recognised as the primary contributor to the pubertal growth spurt in XLH • Shifted focus from standing height to **arm-span length (ASL)** and **sitting height** to better monitor growth in children living with XLH; recognised height may not accurately reflect growth due to bowing of the long bones o An instructional diagram to support HCPs in measuring ASL and SitHt is presented in Fig. 1 • **Collaborated with endocrinologists earlier** to prevent patients being lost to follow-up
Centre 2: UK	• Initiated **early specialist dental referrals** for all patients with XLH, identifying previously undetected dental issues • Organised a presentation at the British Society of Paediatric Dentistry to raise awareness on the importance of early diagnosis, treatment and MDT approach for managing XLH-related dental issues • Incorporated Ear, Nose and Throat (ENT) assessments into clinical evaluations
Centre 3: Bahrain	• Suspected XLH in an adult with lower limb deformities, leg bowing, low serum phosphate and a history of fractures, and confirmed diagnosis through genetic testing o Started burosumab treatment which prevented new fractures • Overcame challenges with burosumab access, resulting in improved patient outcomes for a wheelchair-bound patient • Shared information about the International XLH Alliance with patients and aims to extend its reach to the Gulf Cooperation Council (GCC) region

**Fig. 1:**
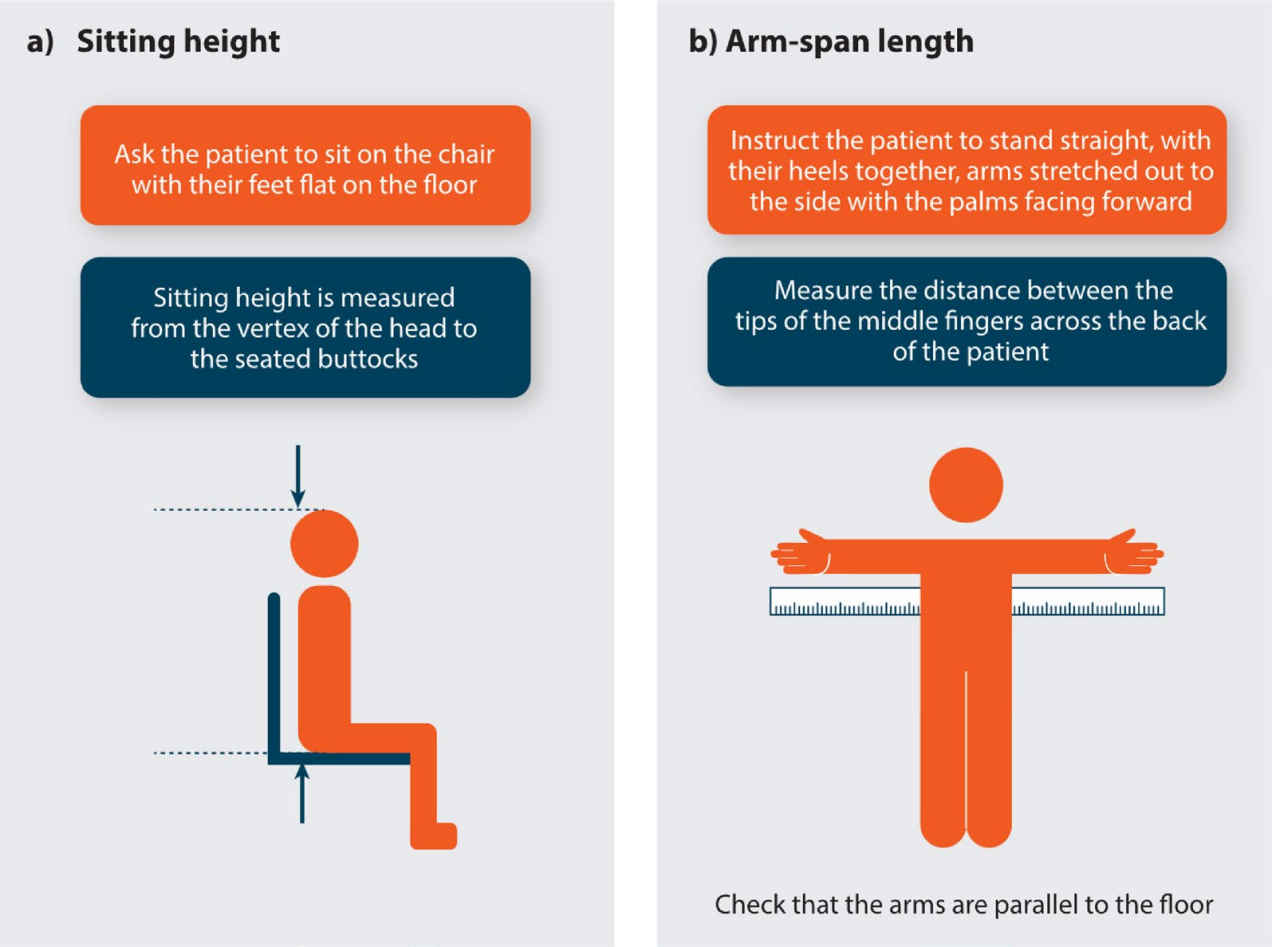
Instructional diagram to support HCPs in measuring: **a** sitting height; **b** arm-span length. *HCP* healthcare professional. Left figure adapted from [56]. Right figure adapted from [57]

For additional information on key aspects of skeletal development and proposed definitions of skeletal growth, please refer to Beck-Nielsen SS, et al. 2021 paper on defining a growing and maturing skeleton [24].

## Role of social media in understanding the patient perspective in XLH

### Social media listening on challenges and support in XLH care

The International XLH Alliance, an umbrella organisation for patient groups affected by XLH, has over 23 global member organisations [25]. In a collaborative effort between the International XLH Alliance and Lumanity, a specialised market research partner in the pharmaceutical industry, a social media listening exercise explored the impact of XLH on individuals and their families. There were three areas of focus for people living with XLH:

(1) identifying challenges faced; (2) outlining primary areas of concern; (3) optimising patient support.

**Fig. 2:**
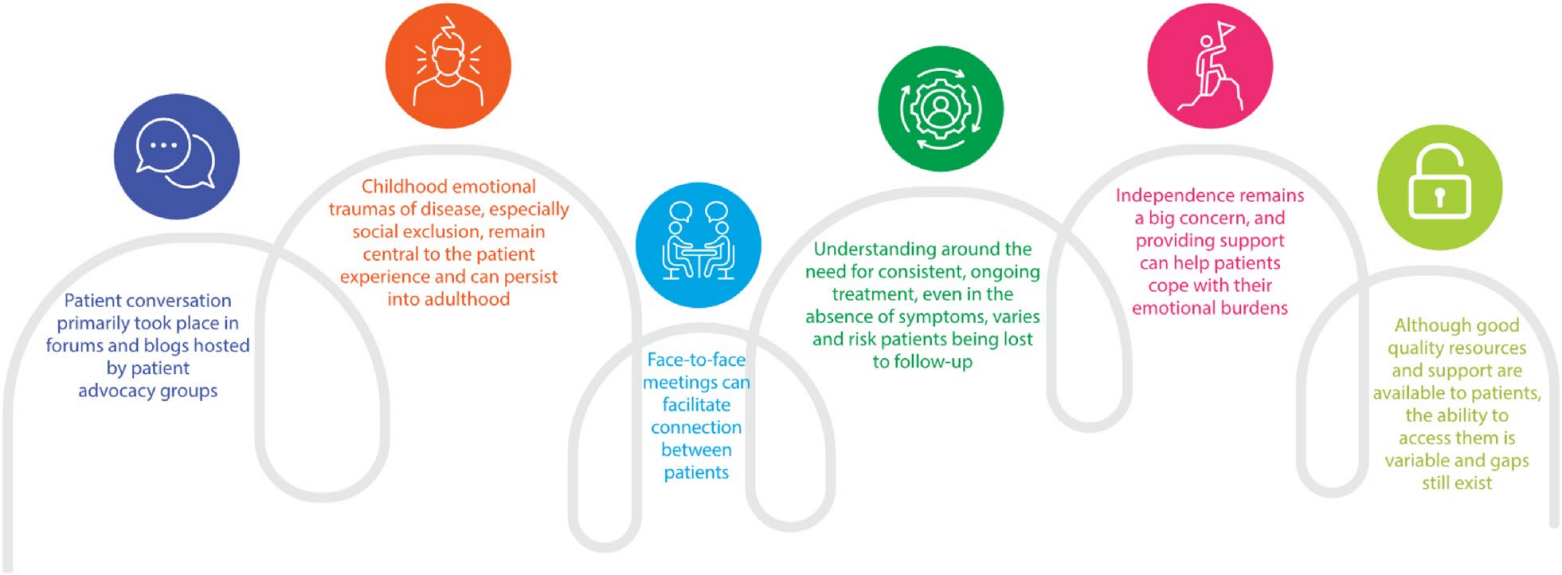
Key findings from the role of social media in understanding the patient perspective of living with XLH. *XLH* X-linked hypophosphataemia

Social media listening software was employed on various platforms over a two-year period to capture communication from people living with XLH. The platforms included: X (formerly Twitter), blogs, Facebook, Instagram and YouTube. Search queries were aimed at people who were talking about a direct patient experience and included XLH terminology, hashtags, treatments and clinical trials. Key findings from the analysis are presented in Fig. 2.

The quality of online information on XLH is generally perceived as high by people living with XLH but they often seek assistance from their peers for diagnosis, interpretation of results, treatment options and emotional support. Support groups, in particular, offer comfort for people living with XLH through social connection. Sharing experiences with peers who understand the challenges of living with XLH provides a sense of solidarity and emotional fortitude.

Positive care experiences specifically include logistical ease of access to specialists and more frequent appointments. Patients also stressed the benefits of face-to-face contact and quick access to ad hoc emergency care. They felt particularly valued and heard by HCPs who communicated openly, and good communication appeared to be a key aspect of seamless transition between specialists. In addition, patients reported confidence in self-advocating with local primary care physicians and comfort in having specialist doctors within their local primary care practices. However, in some countries, people living with XLH encountered more challenging experiences, such as a lack of MDT specialist care, poor communication between HCPs and the burden of coordinating their own care. Young people reported unpleasant encounters with HCPs and experienced difficulties in switching doctors and finding affordable, quality dental care.

Most people living with XLH follow one of two themes when they are reflecting on their journey with XLH (Table 2).

**Table 2:** Key themes in the XLH patient journey

Theme	Description
Theme 1	Majority of young and teenagers with XLH and their caregivers understand the importance of consistent medication, even during periods of disease latency. However, witnessing older relatives experience disease progression can be distressing for younger patients, leading to fears and anxieties about their own future
Theme 2	Some adults misinterpret receding symptoms as a reduction in disease activity, leading them to stop treatment. As a result, the sudden reappearance of symptoms is more shocking and difficult to manage when it occurs

## Collaborating with MDTs for the management of children and adults living with XLH

### XLH care in an MDT setting

**Fig. 3:**
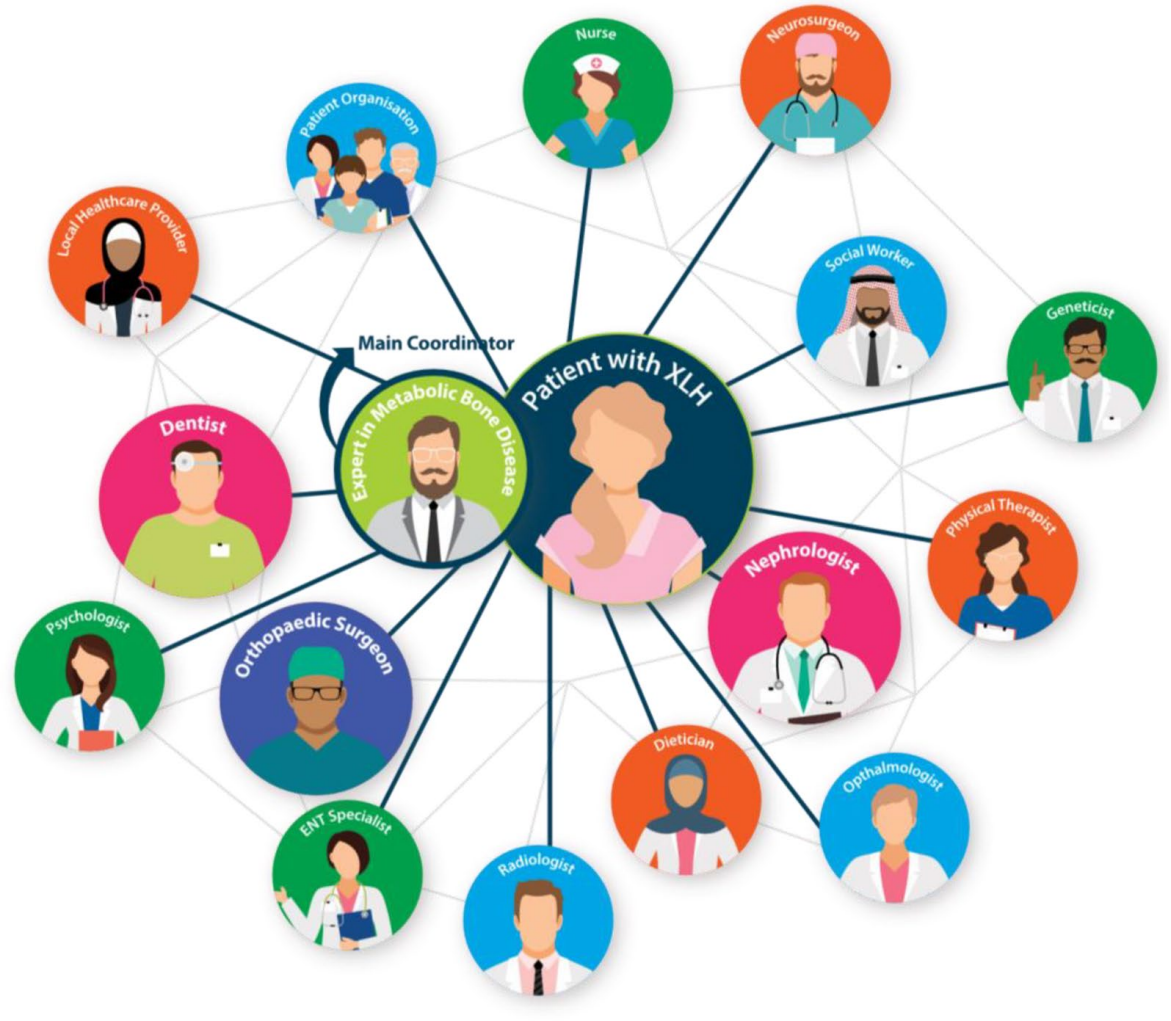
Key MDT members involved in the management of XLH. Larger circles denote the core team while the smaller circles are additional specialties that may be involved depending on individual needs. *ENT* ear, nose and throat, *MDT* multidisciplinary team, *XLH* X-linked hypophosphataemia

Chronic hypophosphataemia associated with XLH has a multisystem impact. Accordingly, people living with XLH need coordinated care by an MDT (Fig. 3) i.e. “a group of healthcare professionals with different areas of expertise who unite to plan and carry out treatment of complex medical conditions” [26].

In the context of XLH, the main coordinator of an MDT is ideally an expert in metabolic bone disease who can:

1. Establish the diagnosis of XLH
2. Initiate and adjust treatment, and follow-up with the patient
3. Perform the initial evaluation and follow-up visits to identify eventual complications of XLH and involve relevant MDT members
4. Organise an individualised management plan based on each patient’s specific clinical manifestations, needs and medical history
5. Take proactive measures to prevent disease-related complications

### Key specialties in the MDT

An overview of MDT organisation and collaboration highlighted the crucial role of specialties in delivering effective XLH care, drawing on clinical experience and current practice recommendations.

### Radiologist

Imaging is an important tool for monitoring disease activity, evaluating therapy-related improvements—such as changes in Rickets Severity Score (RSS)—and detecting disease complications like enthesopathies, as well as treatment-related side effects, including nephrocalcinosis [27]. Radiographic assessments are recommended in children with substantial limb deformities and persistent marked biochemical signs, such as elevated serum alkaline phosphatase (ALP) levels despite adequate therapy [9]. In children and adults with skull morphology in favour of craniosynostosis or clinical signs of increased intracranial pressure, such as persistent headache or vomiting, brain and/or spinal MRI is recommended to exclude craniosynostosis, Chiari 1 malformation or syringomyelia. Renal ultrasonography is the preferred method to screen for nephrocalcinosis [28].

### Orthopaedic surgeon

Orthopaedic surgeons are important in managing the skeletal manifestations of XLH across a patient’s lifetime. In early childhood, limb deformities can represent a major challenge and interfere with physiological motor development. With appropriate timing, guided growth procedures, such as the minimally invasive technique temporary hemiepiphyseodesis, may correct the mechanical leg axis over time. Starting from growth plate fusion and skeletal maturity onwards, correction can only be accomplished using osteotomies. In both instances, mindful consultation and collaboration with the metabolic expert is important to ensure optimal metabolic treatment before any surgical intervention and during the course of healing to avoid suboptimal outcomes and recurrence [29, 30].

Scoliosis, a combined rotational deformity of the spine with lateral curvature, can be associated with XLH, often arising from growth disturbances and reduced vertebral bone stability that compromise spinal alignment. In children and adolescents, lower limb deformities or leg length discrepancies may initially lead to functional scoliosis, which can progress to structural scoliosis over time. Regular follow-up is therefore crucial, with an orthopaedic surgeon playing a key role in monitoring these musculoskeletal manifestations and their potential interactions, ensuring timely and appropriate intervention [31].

Additionally, adults living with XLH should be assessed by an orthopaedic surgeon with expertise in metabolic bone disease. These individuals frequently experience articular cartilage damage and a high prevalence of early-onset osteoarthritis—affecting over 50% of patients by the age of 30, particularly in the hips and knees [32–34]. This highlights the substantial need for orthopaedic consultation that carefully considers the long-term benefits and risks of surgical options. Individualised, timely decision-making should be informed by a comprehensive assessment focused on optimising long-term patient outcomes. While joint replacement surgery can be beneficial in these patients, it should be performed by experienced surgeons [33] familiar with XLH and mindful of the metabolic background to avoid complications.

### Neurosurgeon

Craniosynostosis occurs in approximately 60% of children living with XLH [9]. Chiari malformation type 1, the prolapse of the cerebellar tonsils by 5 mm or more through the foramen magnum, is detected in 25–50% of children living with XLH by use of brain MRI or computed tomography [9]. When these conditions are suspected, an MDT approach involving a neurosurgeon is essential. An ophthalmologist may be required to assess for potential papilledema, while a radiologist, neurosurgeon or craniofacial team should conduct the necessary imaging and evaluations to confirm the diagnosis and plan surgical intervention if required. Additionally, in children with Chiari malformation type 1, a full spinal assessment is crucial to detect any syrinx resulting from cerebrospinal fluid circulation impairment [27].

In adults, chronic back pain, lower extremity weakness and decreased walking distance may be due to spinal stenosis. In such cases, an MRI is recommended, and a neurosurgeon should evaluate the patient for potential benefits of surgery. Headaches, potentially linked to Chiari malformation type 1, also warrant MRI imaging to assess the need for surgical intervention.

### Psychologist

In children and adolescents, mental health issues, including those linked to bullying and body image concerns, should be explored during clinic visits. Quality of life questionnaires and assessments for depression are useful tools for patients to complete. This is particularly important during the teenage years and the transition to adult care, when building trust with a supportive adult—whether a relative, friend or clinician—can help alleviate emotional challenges.

Adults living with XLH may face anxiety or worries about disease progression, feelings of guilt and frustration over limitations in activities of daily living. They should be encouraged to discuss these challenges with trusted family members or friends and referred to a psychologist if mental health issues are suspected. Occupational therapists and social workers can also provide support for managing daily activities and accessing social benefits.

### ENT specialist

The high prevalence of hearing issues among people living with XLH necessitates the involvement of an ENT specialist in the MDT. Reports of hearing loss in patients living with XLH vary significantly based on age and cohort selection criteria. The prevalence ranges from 16% of individuals with hypophosphataemic bone disease experiencing sensorineural hearing loss to 76% of those with X-linked hypophosphataemic osteomalacia identified through pure-tone audiometry [35]. In the latter study, 48% of participants reported experiencing subjective hearing loss [35]. Both children and adults may experience difficulty hearing in environments with significant background noise, understanding conversations on the phone or hearing people when not facing them. Indicators of these include frequently asking others to repeat themselves or perceiving sounds as muffled. In such instances, it is recommended to contact an ENT specialist for a hearing evaluation.

### Key red flags and challenges when collaborating with different specialties in XLH management

Building on the roles outlined previously, attendees at XLH Matters 2024 identified key red flags and challenges requiring prompt specialist referral, along with practical considerations for improving MDT collaboration (summarised in Box 3).

**Box 3:** Understanding the challenges in collaborating with different specialists when managing people living with XLH

*Red flags prompting specialist referral* • *Dental pain*: Recognised by attendees as the primary red flag for both children and adults living with XLH which requires immediate referral to a dentist or orthodontist o *Management tip:* Early and routine dental check-ups, ideally biannually after tooth eruption, are essential to identify and address issues before they escalate. Consider carrying an emergency card for non-specialist odontologists • *Bone deformities and gait abnormalities*: Highlighted as significant indicators for referring children living with XLH to an orthopaedic surgeon o *Management tip:* Effective collaboration requires clear communication about medical treatments and planned surgeries to ensure an integrated and balanced approach • *Fractures/pseudofractures and pain*: Indicated as red flags in adults living with XLH requiring specialised orthopaedic management • *Headaches*: A major red flag for both children and adults, as they may signal intracranial pressure or other neurological complications. Delays in accessing neurological care highlight the importance of establishing efficient referral pathways • *Depression*: Identified as a significant concern in both adults and children living with XLH, necessitating referral to a psychologist and psychiatrist who collaborate as a team. Patient-reported outcome measures (PROMs) can help identify symptoms with psychological impact (Table 3) o *Management tip*: Sharing information on disease severity and patient social background supports effective psychological care *Challenges in specialist collaboration* • *Limited availability and access*: Attendees cited difficulty accessing specialists as a common issue across all disciplines for both children and adults living with XLH • *Timing of interventions*: Coordination of interventions emerged as a major challenge for the attendees in collaborating with orthopaedic surgeons when managing children living with XLH • *Psychological support*: Attendees reported obstacles in collaborating with psychologists when managing adults living with XLH, including access difficulties and uncertainty around screening questionnaire selection and use • *High dental costs*: The expense of dental services creates a collaboration barrier with dentists and orthodontists. Clear treatment plans, follow-up schedules and frequent appointments facilitate proactive dental management

**Table 3:** Selected assessments used for monitoring people living with XLH

Assessment	Measures assessed
EuroQol-5 Dimensions (EQ-5D) (3 versions: 3L, Y-3L, 5L)	Health-related quality of life (HRQoL), covering mobility, self-care, usual activities, pain/discomfort and anxiety/depression [36]
Short-Form 36/SF-36 v2	Different components of physical and mental wellbeing [37]
Patient-Reported Outcomes Measurement Information System (PROMIS)	Pain interference, fatigue and physical function mobility [38]
Western Ontario and McMaster Universities Osteoarthritis Index (WOMAC)	Pain, stiffness and physical function during various activities of daily living [39]
Brief Pain Inventory (BPI)	Intensity and interference of pain [39]

## Expert insights for effective monitoring of children living with XLH

### Scoring radiographs in children living with XLH

Rickets is a key indicator of XLH in paediatric patients, highlighting the importance of its assessment [40]. Standardised radiographic evaluation typically focuses on imaging the knees and wrists to accurately assess the condition.

RSS evaluates metaphyseal fraying, concavity and the proportion of the growth plate affected at various skeletal sites, using a 10-point scale with higher scores indicating more severe rickets. The radiographic response to XLH treatment can be evaluated using the RSS, which correlates with serum ALP levels—a biochemical marker of rickets activity. This correlation provides a comprehensive picture of the patient's condition [30].

**Fig. 4:**
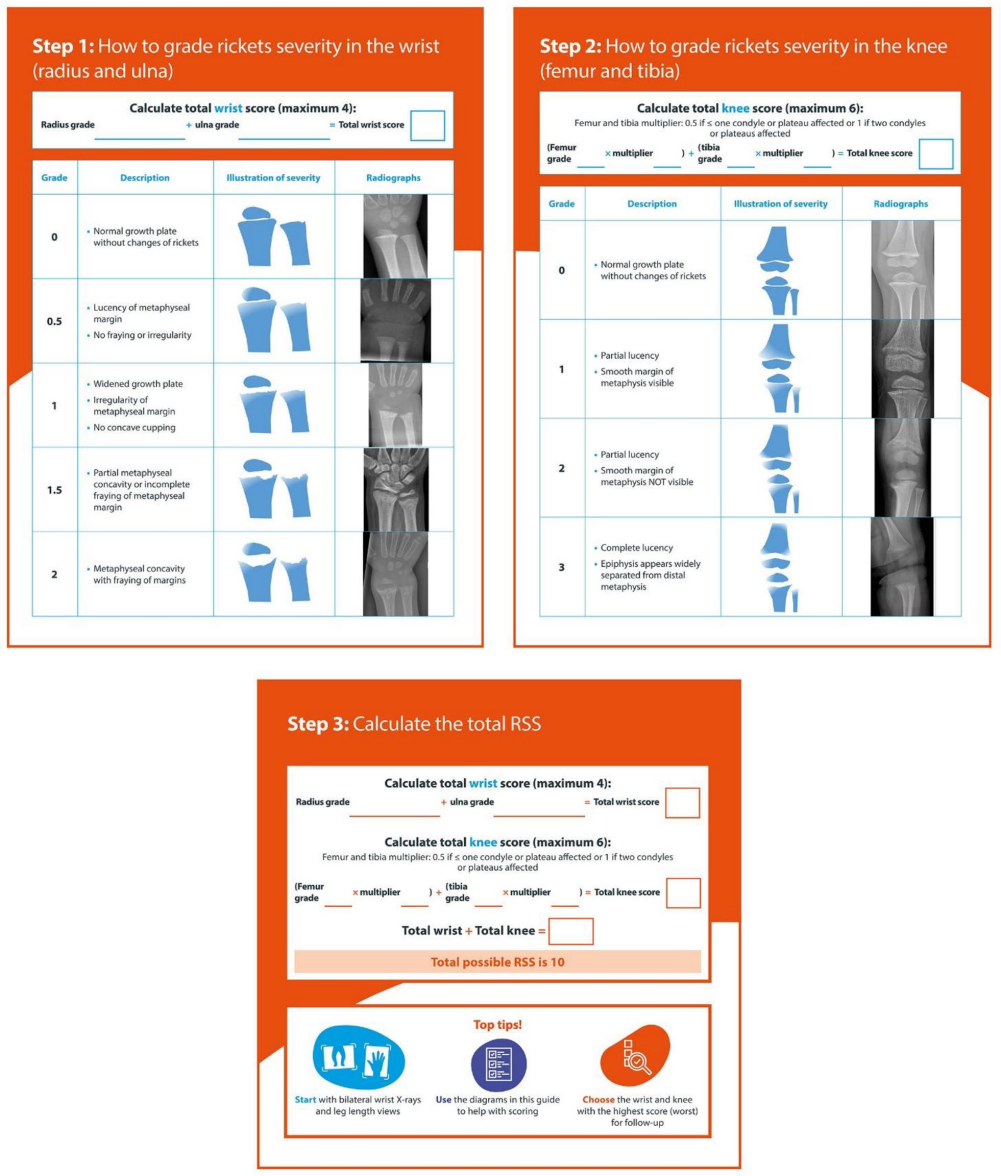
A guide to calculating RSS in children living with XLH. *RSS* Rickets Severity Score. (Adapted from [41] and information courtesy of Dr Rui Santos)

An RSS guide to support clinicians in the quantitative scoring of rickets severity at the wrists and knees was used in an interactive workshop to assess children living with XLH, including assessment of radiographs of real-life case studies (Fig. 4).

RSS calculation has also proven useful to monitor treatment efficacy [41]. The clinical presentation of XLH in children varies but the RSS offers a systematic approach to quantify radiographic abnormality by degree and guide treatment decisions.

### Insights from real-world paediatric case studies

**Fig. 5:**
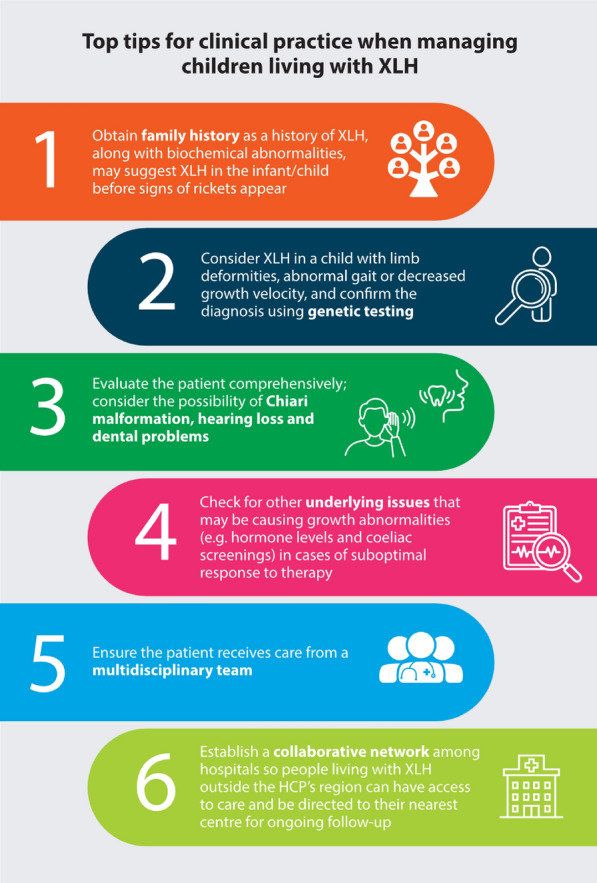
Top tips for clinical practice when managing children living with XLH identified in real-world case studies at XLH Matters 2024. *HCP* healthcare professional, *XLH* X-linked hypophosphataemia

The faculty at XLH Matters 2024 highlighted key considerations in their clinical practice when managing children living with XLH through a series of real-world case studies (Fig. 5).

### Obtaining family history

It is crucial to obtain a family history for infants with suspected XLH because many cases are inherited from an affected family member [5]. Ideally, family history should be assessed before birth, enabling early genetic testing and prompt referral to a specialist team as soon as XLH is suspected. A positive family history of XLH, elevated ALP levels, decreased serum phosphate levels associated with renal phosphate wasting and/or the identification of a *PHEX* mutation can identify affected children within the first weeks of life before clinical signs of rickets become apparent [9]. This is key when considering treatment in infants with XLH as early intervention and management can improve their long-term outcomes.

The primary treatment goal for infants diagnosed with XLH before the onset of bone changes, is to prevent the development of rickets. Serum phosphate levels may fall within the normal range when infants are 3–4 months old, which underscores the importance of evaluating renal phosphate wasting [9]. In people with a negative family history (approximately one-third of reported people living with XLH), genetic analysis of the *PHEX* gene is recommended. This can provide negative or positive confirmation in ~ 70–90% of suspected cases [42].

### Evaluating Chiari malformation, hearing loss and dental problems in children living with XLH

Most children living with XLH who develop craniosynostosis do so within the first few years of life. There are reports of radiological signs of craniosynostosis and scaphocephaly from birth with no noticeable appearance. These cases can remain asymptomatic throughout life but the potential for late-onset complications due to increased intracranial pressure (such as Chiari malformation type 1 and papilloedema) highlights the importance of diagnosing and monitoring craniosynostosis in all children living with XLH [43].

The prevalence of hearing loss varies –﻿ 16–76% depending on age and cohort selection criteria – and has been reported as early as 11 years of age [44]. Hearing evaluations are recommended from the age of 5 years if symptoms of hearing difficulty present [9].

Dental abscesses are very common in XLH and affect up to 70% of children [45]. These children may present with spontaneous endodontic infections on apparently intact teeth, which can be asymptomatic for months or years before evolving into dental abscesses, causing pain and swelling. These abscesses may develop on both deciduous and permanent teeth. If left untreated, endodontic infections and periodontitis can lead to further complications in adulthood, such as tooth loss. Therefore, it is recommended to undergo at least twice-yearly dental examinations after tooth eruption and orthodontic evaluation around the age of 12 years [9].

### Establishing a collaborative network

Ensuring that children living with XLH receive care from an MDT is fundamental for an optimal outcome. Collaborative care between members of the MDT improves XLH management by providing comprehensive evaluations and coordinated treatment plans. Establishing a collaborative network of MDTs among hospitals helps children living with XLH to access specialised care, even if they live outside the immediate region of their HCP. This network facilitates referrals to centres with expertise in managing XLH, maintaining continuity of care and regular follow-ups. Additionally, home assistance programmes can support patients and their families in areas lacking specialised healthcare services, ensuring they receive necessary care and monitoring. Telemedicine also offers a valuable tool for following up with children and adults who live far from tertiary care centres.

## Expert insights for effective monitoring of adults living with XLH

### Key questions and core assessments for monitoring XLH in adults

At XLH Matters 2023, attendees were polled on what assessments they used to monitor adults living with XLH. The results indicated that there was widespread disparity between clinicians in terms of the assessments they regularly used in clinical practice. However, the source of this discrepancy was unclear, therefore, one of the goals of the 2024 meeting was to examine the rationale for these differences. Attendees were asked to identify key questions and assessments for monitoring adults living with XLH.

Assessment of pain was voted as the most pertinent assessment for monitoring adults living with XLH, followed by biochemistry and limitation of movement. The attendees agreed on using the BPI and performing pain assessments at each visit. Measuring patient-reported pain in adults living with XLH can indicate multiple musculoskeletal morbidities, such as osteoarthritis and enthesopathy, as well as fractures/pseudofractures that require further investigation [7]. However, despite the widespread recognition of the importance of assessing pain and biochemistry, attendees at XLH Matters 2024 requested clearer guidance on how to perform common assessments for measuring pain and calculating renal reabsorption of phosphate in people living with XLH.

Guidance on how to use the BPI and calculate the ratio of tubular maximum reabsorption of phosphate to glomerular filtration rate (TmP/GFR), with accompanying reference ranges for adults, is provided in Table 4.

**Table 4:** Recommendations on how to perform the BPI and calculate TmP/GFR in people living with XLH^†^

Assessment	How to use	Reference range
BPI [46]	Begin with the preliminary screening question: "Throughout our lives, most of us have had pain from time to time (such as minor headaches, sprains and toothaches). Have you had pain other than these everyday kinds of pain today?" (Yes/No) Administer the four pain-severity items and seven pain-interference items on a scale of 0–10, where 0 = no interference and 10 = interferes completely • BPI Pain Severity: Assess and score pain at its “worst,” “least,” “average,” and “now” (current pain) and calculate a composite of the four pain items (a mean severity score) • BPI Pain Interference: Calculate the mean of the seven interference items: o General activity o Walking o Work o Mood o Enjoyment of life o Relations with others o Sleep Ensure at least 4 out of 7 items are completed for a valid score	Higher scores indicate worse pain severity or interference
TmP/GFR [47, 48]	$\text{TmP/GFR} = \text{Serum phosphate (mmol/L )} -\left( \, \frac{\text{urine phosphate }\times\text{serum creatinine (mmol/L)}}{\text{urine creatinine }\left(\text{mmol/L}\right)}\right)$	**Both sexes** Newborn: 1.27–2.59 mmol/L 1 month–2 years: 1.15–1.73 mmol/L 2–12 years: 1.22–1.60 mmol/L 12–16 years: 1.09–1.47 mmol/L **Male** 16–25 years: 1.07–1.89 mmol/L 25–45 years: 0.99–1.34 mmol/L 45–65 years: 0.89–1.34 mmol/L 65–75 years: 0.79–1.34 mmol/L **Female** 16–25 years: 1.02–2.05 mmol/L 25–45 years: 0.95–1.42 mmol/L 45–65 years: 0.87–1.40 mmol/L 65–75 years: 0.79–1.34 mmol/L

^†^Use of these outcome measures, in terms of preference, varies depending on clinician, region/country and test availability

*BPI* Brief Pain Inventory, *TmP/GFR* ratio of tubular maximum reabsorption of phosphate to glomerular filtration rate

**Fig. 6:**
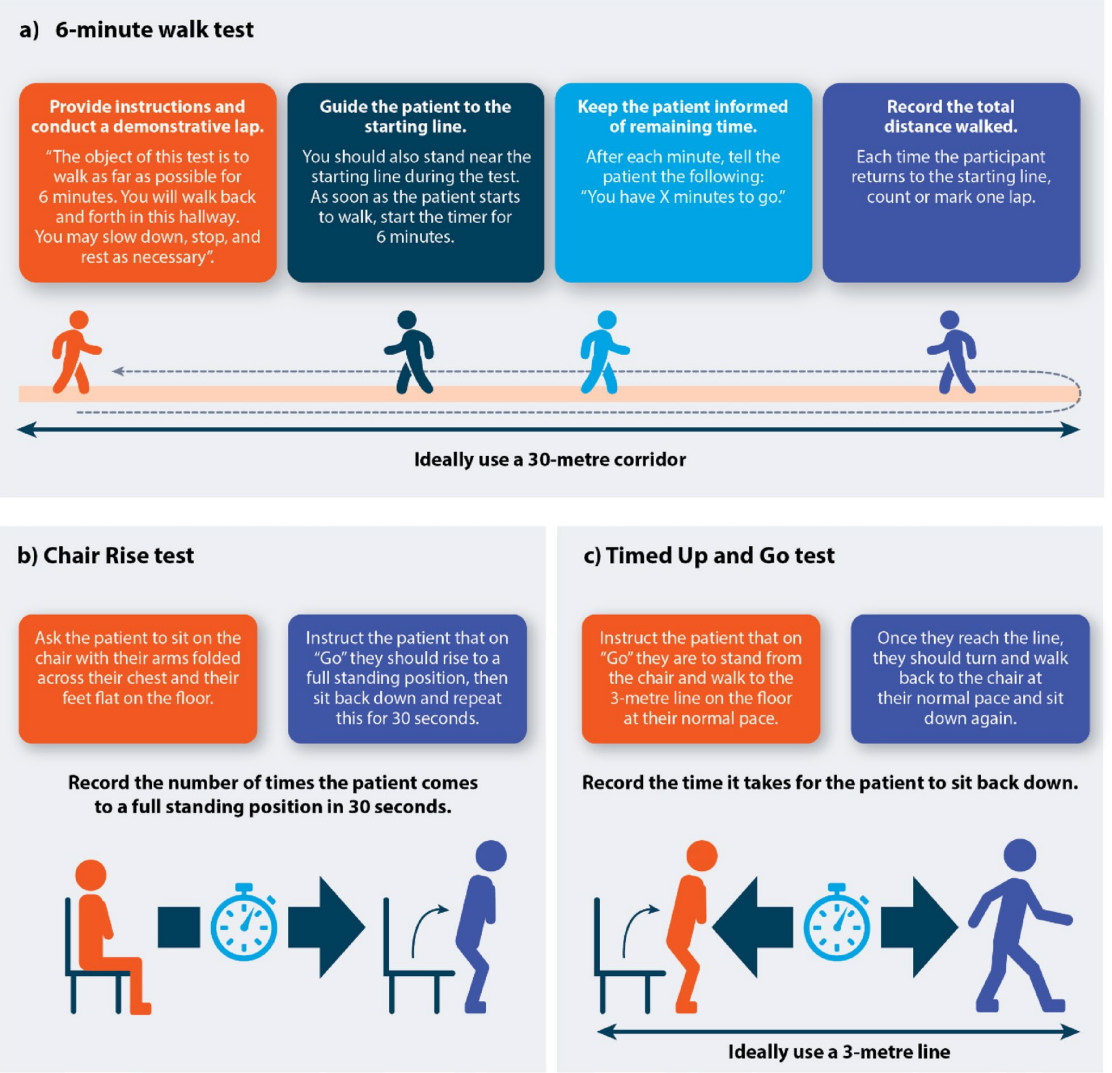
Instructional diagram to support HCPs in performing: **a** the 6MWT; **b** Chair Rise test; **c** the Timed Up and Go test. Adapted from [58–60]. *6MWT* 6-min walk test, *ATS* American Thoracic Society, *CDC* Centers for Disease Control and Prevention, *HCP* healthcare professional

In practice, HCPs can monitor changes in symptoms, function and HRQoL of people living with XLH following treatment using different assessments. Several functional assessments, such as the 6-min walk test (6MWT), Chair Rise test, and the Timed Up and Go test, are used to determine extent of movement in people living with XLH and have also been used in clinical trials to monitor response to burosumab treatment [10, 49, 50]. The advantages of these tests are that they only take a few minutes and minimal resources to complete. An instructional diagram to support HCPs in performing these tests is presented in Fig. 6.

The attendees’ responses on which assessments were essential for monitoring adults living with XLH, exhibited substantial heterogeneity, revealing a wide range of assessments and outcomes that could be used to monitor adults. These findings underscore the multi-faceted nature of XLH and the importance of individualising the assessment of people living with XLH to focus on specific manifestations. Clear guidelines on the gold standard for monitoring adults living with XLH would help standardise assessment and encouraging clinicians and HCPs to share best practices may reduce assessment heterogeneity and accentuate the benefits and limitations of different monitoring strategies.

### Insights from real-world adult case studies

**Fig. 7:**
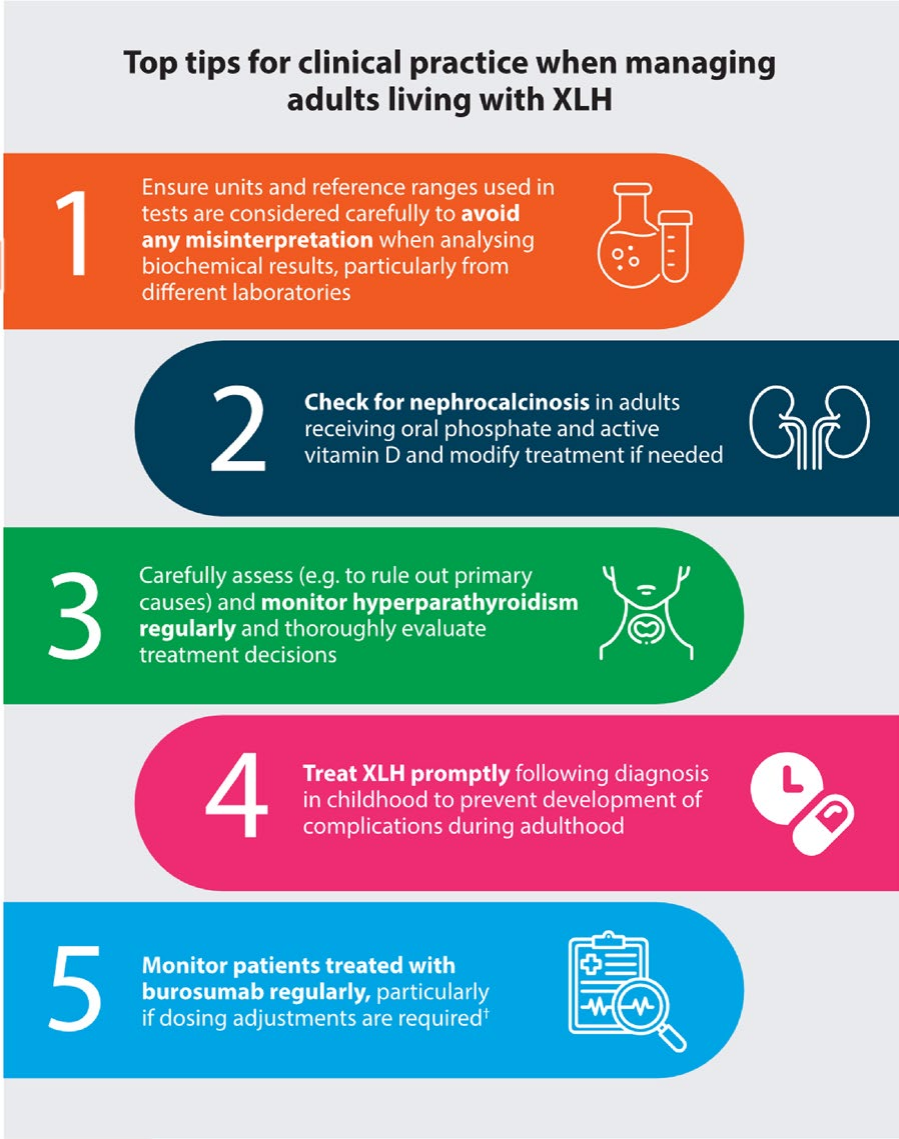
Top tips for clinical practice when managing adults living with XLH identified in real-world case studies at XLH Matters 2024. ^†^Please refer to the Summary of Product Characteristics of burosumab for more information on dosing. *XLH* X-linked hypophosphataemia

A series of real-world adult case studies discussed during the meeting outlined important considerations that clinicians have faced when managing adults living with XLH in their clinical practice (Fig. 7).

### Checking for nephrocalcinosis in XLH

Nephrocalcinosis, nephrolithiasis and impaired renal function occur as complications related to oral phosphate and active vitamin D. Development of nephrocalcinosis is associated with elevated urinary calcium and phosphate levels, which can also lead to the development of nephrolithiasis [51]. It is, therefore, essential to monitor nephrocalcinosis in people receiving oral phosphate and active vitamin D and modify treatment, if needed, to manage these complications.

### Promptly treating XLH following diagnosis

Prompt treatment following diagnosis of XLH in childhood can help to prevent the progression of clinical manifestations in adulthood [8]. A group of European experts convened in 2020 to share best practice on the use of burosumab in children and adolescents living with XLH [52]. The consensus was to start burosumab treatment as early as possible in children living with XLH aged ≥ 1 year, particularly in those with RSS ≥ 2 [52]. This consensus aligned with a group of eight global experts in XLH who reviewed the long-term effects of burosumab on clinical sequelae. Starting treatment with burosumab in childhood and continuing throughout adulthood to maintain normal serum phosphate levels, may optimise patient outcomes and increase the likelihood of an aligned skeleton, however, further real-world data are required to support this [53]. These insights were echoed by attendees and faculty members at XLH Matters 2024, indicating widespread agreement on the importance of starting burosumab treatment early in children living with XLH.

## Future outlook for the management of XLH

At XLH Matters 2024, the different aspects of current treatment practices in XLH were explored. The recommendations discussed in this section are informed by evidence from the latest clinical research and the upcoming guidelines for XLH management [28]. Audience polling revealed variability in defining a satisfactory response to either oral phosphate and active vitamin D or burosumab, in both children and adults living with XLH. This variability draws attention to potential gaps in knowledge or consensus regarding the management of XLH and highlights the need for updated, standardised guidelines to incorporate recent data on burosumab and ensure uniformity in evaluating treatment efficacy.

### Optimising treatment in children living with XLH

There are different approaches to determine a satisfactory treatment response to either oral phosphate and active vitamin D or burosumab in children living with XLH. European guidelines on the diagnosis and management of XLH recommend that a satisfactory response to oral phosphate and active vitamin D in children living with XLH may be defined as a significant improvement in rickets activity, bone pain, serum ALP and radiological rickets severity, within 12 months of treatment after an initial improvement in ALP levels. By 24 months, significant improvement in leg deformities and normal growth velocity (> 25th percentile for sex and age) may also be evident [28].

European guidelines recommend that burosumab should be initiated as soon as XLH diagnosis is established in children and adolescents (aged 1–17 years) who present with signs of rickets, such as leg deformities, elevated total ALP and/or radiological evidence. For children not eligible for burosumab (e.g. those under 12 months of age) or when burosumab is unavailable, oral phosphate and active vitamin D should be started [28].

Transition from oral phosphate and active vitamin D to burosumab may be necessary if there is an insufficient skeletal response, significant side effects (such as severe nephrocalcinosis, persistent gastrointestinal discomfort, diarrhoea, hyperparathyroidism or hypercalcaemia), poor adherence to treatment or persistent short stature, as recommended by the guidelines [28].

According to the European guidelines, a satisfactory treatment response to burosumab in children includes significant improvement in renal phosphate wasting, serum phosphate levels and rickets activity, including bone pain and ALP levels, which may be observed within 6 months of starting treatment. By 24 months, progressive improvement in leg deformities, ALP values within the normal range for age and normal growth velocity (> 25th percentile for sex and age) can be expected [28].

The recommended starting dose for burosumab in children and adolescents living with XLH (1–17 years) is 0.8 mg/kg of body weight given every 2 weeks, rounded to the nearest 10 mg, up to a maximum dose of 90 mg [54]. The European guidelines recommend maintaining the dose if serum phosphate levels and rickets activity significantly improve within 3–6 months or if leg deformities progressively improve and ALP levels are normalised within 24 months, even if serum phosphate levels remain below the age-related lower normal range [28]. In the long-term, tailoring burosumab treatment in children may be guided by clinical and biochemical improvement of rickets rather than by serum phosphate levels alone. In cases where the response to burosumab is deemed insufficient, assessing for calcium and vitamin D deficiency, hyperparathyroidism, adherence issues, incorrect dosage administration and inadequate self-administration technique are recommended. If there is uncertainty regarding a satisfactory response to burosumab, radiological assessment for rickets activity is recommended [28].

During the transition from paediatric to adult healthcare services, the European guidelines suggest continuing burosumab, if available after cessation of growth, until at least the middle of the third decade, which is the time of peak bone mass [28]. If burosumab is not available, treatment with oral phosphate and active vitamin D is suggested. Continued use of burosumab beyond skeletal maturity may preserve or enhance bone mineralisation, reduce osteomalacia, significantly impact the long-term consequences of XLH and improve overall quality-of-life throughout a patient’s lifespan [28, 55]. However, it is important to note that availability of burosumab treatment after the end of skeletal growth varies across countries.

### Optimising treatment in adults living with XLH

The European guidelines propose defining a satisfactory response to oral phosphate and active vitamin D in adults as significant improvement in signs of osteomalacia (such as radiological lesions and pseudofractures), musculoskeletal pain, stiffness and ALP levels within 24 months of treatment [28]. A satisfactory response with burosumab may be defined as significant improvement in renal phosphate wasting, serum phosphate levels and musculoskeletal pain within 6 months, with expected further improvements in pain, stiffness, signs of osteomalacia and ALP levels by 24 months [28].

The European guidelines suggest oral phosphate and active vitamin D may be initiated in adults if there are biochemical or clinical signs of osteomalacia, musculoskeletal pain or stiffness, or if burosumab is contraindicated, such as during pregnancy [28]. However, oral phosphate and active vitamin D treatment is associated with side effects such as severe nephrocalcinosis, persistent gastrointestinal discomfort and/or hyperparathyroidism. In such cases, or when pseudofractures are present or there is an insufficient musculoskeletal response, switching to burosumab can offer benefits [28].

Routine treatment of adults living with XLH requires a thorough clinical assessment as many patients reduce activity levels to manage their symptoms. In the European guidelines, treatment is indicated when symptoms such as pseudofractures, musculoskeletal pain, stiffness, or abnormalities such as elevated ALP or radiological signs of osteomalacia are present, after ruling out other causes. Furthermore, treatment should be considered if orthopaedic or dental implant surgery is planned [28].

The recommended starting dose of burosumab in adults living with XLH is 1 mg/kg of body weight, rounded to the nearest 10 mg, up to a maximum dose of 90 mg, given every 4 weeks [54]. If the response to burosumab treatment is insufficient, the guidelines recommend increasing the dose of burosumab to a maximum of 90 mg while aiming to keep serum phosphate levels below the upper limit of normal [28].

## Conclusion

The XLH Matters 2024 meeting highlighted key advancements in the understanding and management of XLH, reflecting ongoing progress in the field. These developments enhance clinical practice, from improved diagnostic approaches and treatment strategies to better interdisciplinary care. The integration of social media insights revealed gaps in patient support, especially for adults, and stressed the role of advocacy groups in enriching care.

Discussions at the meeting also emphasised the need for MDT management, regular assessments and utilisation of tools, such as the RSS, for monitoring children. In adults, the focus was on pain evaluation, the use of PROMs and early treatment to prevent disease progression. Looking ahead, the upcoming 2025 guidelines for the management of XLH will offer standardised frameworks to improve care, ultimately enhancing patient outcomes and quality of life.

## Data Availability

No datasets used in this supplement.

## Abbreviations

1,25(OH)_2_D: 1,25-Dihydroxyvitamin D (calcitriol)
6MWT: 6‑Minute walk test
ALP: Alkaline phosphatase
ASL: Arm-span length
BPI: Brief Pain Inventory
ENT: Ear, Nose and Throat
EQ-5D (3L, Y-3L, 5L): EuroQol-5 Dimensions (3 level, child‑friendly‑3 level, 5 level)
FGF23: Fibroblast growth factor 23
GCC: Gulf Cooperation Council
HCP: Healthcare professional
HRQoL: Health-related quality of life
MDT: Multidisciplinary team
MRI: Magnetic resonance imaging
PCr/Pi: Phosphocreatine to inorganic phosphate ratio
PHEX: Phosphate-regulating endopeptidase homologue on the X chromosome
PROM: Patient-reported outcome measure
PROMIS: Patient-Reported Outcomes Measurement Information System
Q2W: Every 2 weeks
RSS: Rickets Severity Score
SD: Standard deviation
SF-36: Short-Form 36
SH/H: Sitting height/standing height
TmP/ GFR: Ratio of tubular maximum reabsorption of phosphate to glomerular filtration rate
TRP: Tubular reabsorption of phosphate
WOMAC: Western Ontario and McMaster Universities Osteoarthritis Index
WT: Wildtype
XLH: X‑linked hypophosphataemia

## References

[1] Francis F, et al. A gene (PEX) with homologies to endopeptidases is mutated in patients with X-linked hypophosphatemic rickets. Nat Genet. 1995;11:130–6. 7550339 10.1038/ng1095-130

[2] Rush ET, et al. Molecular diagnoses of X-linked and other genetic hypophosphatemias: results from a sponsored genetic testing program. J Bone Miner Res. 2022;37:202–14. 34633109 10.1002/jbmr.4454 PMC9298723

[3] Ichikawa S, et al. Mutational survey of the *PHEX* gene in patients with X-linked hypophosphatemic rickets. Bone. 2008;43:663–6. 18625346 10.1016/j.bone.2008.06.002 PMC2579265

[4] Sant’ Ana I, et al. X-linked hypophosphatemic rickets: description of seven new variants in patients followed up in reference hospitals in Rio de Janeiro. Mol Genet Genomic Med. 2022;10: e1941. 35384411 10.1002/mgg3.1941 PMC9184672

[5] Raimann A, et al. Multidisciplinary patient care in X-linked hypophosphatemic rickets: one challenge, many perspectives. Wien Med Wochenschr. 2020;170:116–23. 31993875 10.1007/s10354-019-00732-2 PMC7098922

[6] Rajah J, et al. Clinical practice: diagnostic approach to the rachitic child. Eur J Pediatr. 2011;170:1089–96. 21833499 10.1007/s00431-011-1529-z

[7] Cheung M, et al. Patient-reported complications, symptoms, and experiences of living with X-linked hypophosphatemia across the life-course. J Endocr Soc. 2021;5:bvab070. 34258488 10.1210/jendso/bvab070 PMC8272533

[8] Skrinar A, et al. The lifelong impact of X-linked hypophosphatemia: Results from a burden of disease survey. J Endocr Soc. 2019;3:1321–34. 31259293 10.1210/js.2018-00365 PMC6595532

[9] Haffner D, et al. Clinical practice recommendations for the diagnosis and management of X-linked hypophosphataemia. Nat Rev Nephrol. 2019;15:435–55. 31068690 10.1038/s41581-019-0152-5 PMC7136170

[10] Kamenicky P, et al. Benefit of burosumab in adults with X-linked hypophosphataemia (XLH) is maintained with long-term treatment. RMD Open. 2023;9: e002676. 36854566 10.1136/rmdopen-2022-002676 PMC9980374

[11] Javaid MK, et al. Musculoskeletal features in adults with X-linked hypophosphatemia: an analysis of clinical trial and survey data. J Clin Endocrinol Metab. 2022;107:e1249–62. 34636401 10.1210/clinem/dgab739 PMC8852215

[12] Seefried L, et al. XLH Matters 2022: insights and recommendations to improve outcomes for people living with X-linked hypophosphataemia (XLH). Orphanet J Rare Dis. 2023;18(Suppl 2):333. 37885021 10.1186/s13023-023-02883-3 PMC10604503

[13] Seefried L, et al. XLH Matters: an evolving programme to discuss new advances and share clinical experiences to improve patient outcomes. Orphanet J Rare Dis. 2025;19(Suppl 2):497. 39901153 10.1186/s13023-024-03387-4 PMC11792233

[14] Baroncelli GI, et al. Intact FGF23 concentration in healthy infants, children, and adolescents, and diagnostic usefulness in patients with X-linked hypophosphatemic rickets. J Endocrinol Investig. 2024;47:873–82. 37991698 10.1007/s40618-023-02202-4 PMC10965647

[15] Carpenter TO, et al. Burosumab therapy in children with X-linked hypophosphatemia. N Engl J Med. 2018;378:1987–98. 29791829 10.1056/NEJMoa1714641

[16] Ward LM, et al. Burosumab vs conventional therapy in children with X-linked hypophosphatemia: results of the open-label, phase 3 extension period. JBMR Plus. 2024;8:ziad001. 38690124 10.1093/jbmrpl/ziad001 PMC11059996

[17] Imel EA, et al. Burosumab versus conventional therapy in children with X-linked hypophosphataemia: a randomised, active-controlled, open-label, phase 3 trial. Lancet. 2019;393:2416–27. 31104833 10.1016/S0140-6736(19)30654-3 PMC7179969

[18] de Tienda M, et al. MRI quantitative muscle characterization in children with X-linked hypophosphatemia. Orthop Traumatol Surg Res. 2024;11: 103713. 10.1016/j.otsr.2023.103713 37863188

[19] Kara JAS, et al. Impaired physical performance in X-linked hypophosphatemia is not caused by depleted muscular phosphate stores. J Clin Endocrinol Metab. 2023;108:1634–45. 37043477 10.1210/clinem/dgad210

[20] Boros E, et al. Adult height improved over decades in patients with X-linked hypophosphatemia: a cohort study. Eur J Endocrinol. 2023;189:469–75. 37831782 10.1093/ejendo/lvad144

[21] Lira dos Santos EJ, et al. Dental impact of anti-fibroblast growth factor 23 therapy in X-linked hypophosphatemia. Int J Oral Sci. 2023;15:53. 38052774 10.1038/s41368-023-00259-8 PMC10697996

[22] Rana R, et al. Impaired 1,25-dihydroxyvitamin D_3_ action underlies enthesopathy development in the *Hyp* mouse model of X-linked hypophosphatemia. JCI Insight. 2023;8: e163259. 37490334 10.1172/jci.insight.163259 PMC10544216

[23] Del Pino M, et al. Growth in height and body proportion from birth to adulthood in hereditary hypophosphatemic rickets: a retrospective cohort study. J Endocrinol Investig. 2022;45:1349–58. 35226335 10.1007/s40618-022-01768-9

[24] Beck-Nielsen SS, et al. Defining a growing and maturing skeleton and its relevance in diseases that affect skeletal growth, such as X-linked hypophosphataemia (XLH). Int J Rare Dis Disord. 2021;4:029.

[25] International XLH Alliance. Available at: https://xlhalliance.org/. Accessed: July 2025.

[26] Concise Medical Dictionary (8 ed.), Oxford University Press 2014. Available at: https://www.oxfordreference.com/display/10.1093/oi/authority.20110803100215626. Accessed: July 2025.

[27] Adamsbaum C, et al. Contribution of imaging to the diagnosis and follow up of X-linked hypophosphatemia. Arch Pediatr. 2021;28:594–8. 34583869 10.1016/j.arcped.2021.09.002

[28] Haffner D, et al. Clinical practice recommendations for the diagnosis and management of X-linked hypophosphataemia. Nat Rev Nephrol. 2025. 10.1038/s41581-024-00926-x. 39814982 10.1038/s41581-024-00926-x

[29] Gizard A, et al. Outcomes of orthopedic surgery in a cohort of 49 patients with X-linked hypophosphatemic rickets (XLHR). Endocr Connect. 2017;6:566–73. 28954742 10.1530/EC-17-0154 PMC5633063

[30] Baroncelli GI, Mora S. X-linked hypophosphatemic rickets: multisystemic disorder in children requiring multidisciplinary management. Front Endocrinol (Lausanne). 2021;12: 688309. 34421819 10.3389/fendo.2021.688309 PMC8378329

[31] Higuchi C. Orthopedic complications and management in children with X-linked hypophosphatemia. Endocrines. 2022;3:488–97.

[32] Macica CM, Tommasini SM. Biomechanical impact of phosphate wasting on articular cartilage using the murine Hyp model of X-linked hypophosphatemia. JBMR Plus. 2023;7: e10796. 37808393 10.1002/jbm4.10796 PMC10556269

[33] Mills ES, et al. Joint replacement in X-linked hypophosphatemia. J Orthop. 2018;16:55–60. 30662239 10.1016/j.jor.2018.12.007 PMC6324762

[34] Seefried L, et al. Burden of disease associated with X-linked hypophosphataemia in adults: a systematic literature review. Osteoporos Int. 2021;32:7–22. 32710160 10.1007/s00198-020-05548-0 PMC7755619

[35] Beck-Nielsen SS, et al. FGF23 and its role in X-linked hypophosphatemia-related morbidity. Orphanet J Rare Dis. 2019;14:58. 30808384 10.1186/s13023-019-1014-8 PMC6390548

[36] EUROQOL. EQ-5D-5L. Available at: https://euroqol.org/information-and-support/euroqol-instruments/eq-5d-5l/. Accessed: July 2025.

[37] Lins L, Carvalho FM. SF-36 total score as a single measure of health-related quality of life: scoping review. SAGE Open Med. 2016;4:2050312116671725. 27757230 10.1177/2050312116671725 PMC5052926

[38] Padidela R, et al. Patient-reported outcomes from a randomized, active-controlled, open-label, phase 3 trial of burosumab versus conventional therapy in children with X-linked hypophosphatemia. Calcif Tissue Int. 2021;108:622–33. 33484279 10.1007/s00223-020-00797-x PMC8064984

[39] Ito N, et al. Burden of disease of X-linked hypophosphatemia in Japanese and Korean patients: a cross-sectional survey. Endocr J. 2022;69:373–83. 34732603 10.1507/endocrj.EJ21-0386

[40] Park E, Kang HG. X-linked hypophosphatemic rickets: from diagnosis to management. Clin Exp Pediatr. 2024;67:17–25. 37321578 10.3345/cep.2022.01459 PMC10764665

[41] Thacher TD, et al. Rickets severity predicts clinical outcomes in children with X-linked hypophosphatemia: utility of the radiographic Rickets Severity Score. Bone. 2019;122:76–81. 30772600 10.1016/j.bone.2019.02.010

[42] Giannini S, et al. Burden of disease and clinical targets in adult patients with X-linked hypophosphatemia. A comprehensive review. Osteoporos Int. 2021;32:1937–49. 34009447 10.1007/s00198-021-05997-1 PMC8510985

[43] Munns CF, et al. Craniosynostosis in patients with X-linked hypophosphatemia: a review. JBMR Plus. 2023;7: e10728. 37197318 10.1002/jbm4.10728 PMC10184010

[44] Dahir K, et al. X-linked hypophosphatemia: a new era in management. J Endocr Soc. 2020;4:bvaa151. 33204932 10.1210/jendso/bvaa151 PMC7649833

[45] Biosse-Duplan M, et al. Dental and periodontal features and management in XLH children and adults. Int J Bone Frag. 2021;1:74–9.

[46] Cleeland CS. The Brief Pain Inventory User Guide. Available at: https://www.mdanderson.org/documents/Departments-and-Divisions/Symptom-Research/BPI_UserGuide.pdf. Accessed: July 2025.

[47] Stark H, et al. Direct measurement of TP/GFR: a simple and reliable parameter of renal phosphate handling. Nephron. 1986;44:125–8. 3774075 10.1159/000184216

[48] Chong WH, et al. Tumor-induced osteomalacia. Endocr Relat Cancer. 2011;18:R53-77. 21490240 10.1530/ERC-11-0006 PMC3433741

[49] Insogna KL, et al. A randomized, double-blind, placebo-controlled, Phase 3 trial evaluating the efficacy of burosumab, an anti-FGF23 antibody, in adults with X-linked hypophosphatemia: week 24 primary analysis. J Bone Miner Res. 2018;33:1383–93. 29947083 10.1002/jbmr.3475

[50] Briot K, et al. Burosumab treatment in adults with X-linked hypophosphataemia: 96-week patient-reported outcomes and ambulatory function from a randomised phase 3 trial and open-label extension. RMD Open. 2021;7: e001714. 34548383 10.1136/rmdopen-2021-001714 PMC8458321

[51] Harada D, et al. Switching from conventional therapy to burosumab injection has the potential to prevent nephrocalcinosis in patients with X-linked hypophosphatemic rickets. J Pediatr Endocrinol Metab. 2021;34:791–8. 33837680 10.1515/jpem-2020-0734

[52] Mughal MZ, et al. Burosumab for X-linked hypophosphatemia in children and adolescents: opinion based on early experience in seven European countries. Front Endocrinol (Lausanne). 2023;13:1034580. 36798486 10.3389/fendo.2022.1034580 PMC9928183

[53] Seefried L, et al. Anticipated effects of burosumab treatment on long-term clinical sequelae in XLH: expert perspectives. Front Endocrinol (Lausanne). 2023;14:1211426. 37547321 10.3389/fendo.2023.1211426 PMC10400326

[54] Kyowa Kirin Limited. CRYSVITA (burosumab). Summary of Product Characteristics.

[55] Glorieux FH, et al. Potential influences on optimizing long-term musculoskeletal health in children and adolescents with X-linked hypophosphatemia (XLH). Orphanet J Rare Dis. 2022;17:30. 35101067 10.1186/s13023-021-02156-x PMC8802511

[56] Bogin B, Varela-Silva MI. Leg length, body proportion, and health: a review with a note on beauty. Int J Environ Res Public Health. 2010;7:1047–75. 20617018 10.3390/ijerph7031047 PMC2872302

[57] Tsuji M, et al. Comparative study on three different methods for arm-span measurement: the Japan environment and Children’s study pilot. Environ Health Prev Med. 2017;22:28. 29165129 10.1186/s12199-017-0632-9 PMC5664793

[58] ATS Committee on Proficiency Standards for Clinical Pulmonary Function Laboratories. ATS statement: guidelines for the six-minute walk test. Am J Respir Crit Care Med. 2002;166:111–7. 12091180 10.1164/ajrccm.166.1.at1102

[59] Centers for Disease Control and Prevention. Assessment 30-second chair stand. 2017. Available at: https://www.cdc.gov/steadi/media/pdfs/STEADI-Assessment-30Sec-508.pdf. Accessed: July 2025.

[60] Centers for Disease Control and Prevention. Assessment timed up and go. 2017. Available at: https://www.cdc.gov/steadi/media/pdfs/STEADI-Assessment-TUG-508.pdf. Accessed: July 2025.

